# Prenatal overexpression of platelet‐derived growth factor receptor A results in central nervous system hypomyelination

**DOI:** 10.1002/brb3.2332

**Published:** 2021-09-04

**Authors:** Herminio Joey Cardona, Agila Somasundaram, Donna M. Crabtree, Samantha L. Gadd, Oren J. Becher

**Affiliations:** ^1^ Division of Hematology Oncology, Neuro‐Oncology, and Stem Cell Transplant, Ann & Robert H. Lurie Children's Hospital Chicago Illinois USA; ^2^ Department of Pediatrics Duke University Medical Center Durham North Carolina USA; ^3^ Office of Clinical Research, Duke University Medical Center Durham NC USA; ^4^ Department of Pathology Ann & Robert H. Lurie Children's Hospital Chicago Illinois USA; ^5^ Department of Pediatrics Northwestern University Chicago Illinois USA; ^6^ Department of Biochemistry and Molecular Genetics Northwestern University Chicago Illinois USA

**Keywords:** brain tumors, diffuse midline glioma, hypomyelination, myelination, oligodendrocyte precursor cells, PDGF, PDGFRA

## Abstract

**Background:**

Platelet‐derived growth factor (PDGF) signaling, through the ligand PDGF‐A and its receptor PDGFRA, is important for the growth and maintenance of oligodendrocyte progenitor cells (OPCs) in the central nervous system (CNS). PDGFRA signaling is downregulated prior to OPC differentiation into mature myelinating oligodendrocytes. By contrast, PDGFRA is often genetically amplified or mutated in many types of gliomas, including diffuse midline glioma (DMG) where OPCs are considered the most likely cell‐of‐origin. The cellular and molecular changes that occur in OPCs in response to unregulated PDGFRA expression, however, are not known.

**Methods:**

Here, we created a conditional knock‐in (KI) mouse that overexpresses wild type (WT) human PDGFRA (hPDGFRA) in prenatal Olig2‐expressing progenitors, and examined in vivo cellular and molecular consequences.

**Results:**

The KI mice exhibited stunted growth, ataxia, and a severe loss of myelination in the brain and spinal cord. When combined with the loss of p53, a tumor suppressor gene whose activity is decreased in DMG, the KI mice failed to develop tumors but still exhibited hypomyelination. RNA‐sequencing analysis revealed decreased myelination gene signatures, indicating a defect in oligodendroglial development. Mice overexpressing PDGFRA in prenatal GFAP‐expressing progenitors, which give rise to a broader lineage of cells than Olig2‐progenitors, also developed myelination defects.

**Conclusion:**

Our results suggest that embryonic overexpression of hPDGFRA in Olig2‐ or GFAP‐progenitors is deleterious to OPC development and leads to CNS hypomyelination.

## Graphical abstract

A novel PDGFRA knock‐in mouse was developed, and it was noted that PDGFRA overexpression in prenatal OPCs results in CNS hypomyelination. Furthermore, PDGFRA overexpression in prenatal OPCs along with p53 loss does not induce gliomas.

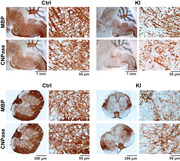

## 1 INTRODUCTION

The proliferation and survival of oligodendrocyte progenitor cells (OPCs) are dependent on signaling through platelet‐derived growth factor A (PDGF‐A) and its receptor PDGFRA (Hart et al., 1989; Pringle et al., 1989; Richardson et al., 1988). PDGFRA signaling, however, is inhibitory to OPC differentiation and is downregulated prior to OPC maturation into myelinating oligodendrocytes (OLs) (Calver et al., 1998; Richardson et al., 1988). Removing PDGF‐A from cell culture medium induces OPCs to differentiate into OLs (Hall et al., 1996), and PDGFRA is expressed in proliferating OPCs, but lost in myelinating OLs (Butt, Hornby, Ibrahim, et al., 1997; Ellison & de Vellis, 1994). PDGFRA downregulation during OPC differentiation occurs through transcriptional, translational and/or post‐translational mechanisms (Calabretta et al., 2018; Dugas et al., 2010; Zhao et al., 2010; Zhu et al., 2014). The transcription factor Nkx2.2 directly binds the promoter region of *PDGFRA* and inhibits its expression during OPC differentiation (Zhu et al., 2014). MicroRNAs miR‐219 and miR‐338 are upregulated during OPC differentiation and repress PDGFRA transcripts by binding the 3′ untranslated region (Dugas et al., 2010; Zhao et al., 2010). Progressive loss of the methyltransferase PRMT5 facilitates ubiquitination and degradation of membrane PDGFRA thereby promoting OPC differentiation (Calabretta et al., 2018).

PDGFRA signaling, on the other hand, is frequently elevated in adult and pediatric gliomas (Mackay et al., 2017), including the rare and universally fatal diffuse midline glioma (DMG), which is predominantly found in pediatric patients (Buczkowicz & Hawkins, 2015). Amplifications of the wild type (WT) PDGFRA gene locus have been found in approximately 12% of adult and 8–39% of pediatric cases, and activating mutations in approximately 13% of adult and 4–9% of pediatric cases, respectively (Brennan et al., 2013; Buczkowicz & Hawkins, 2015; Paugh et al., 2011; Puget et al., 2012). Moreover, emerging evidence suggests that neonatal OPCs are a likely cell‐of‐origin for DMG (Filbin et al., 2018; Lindquist et al., 2016; Nagaraja et al., 2017). Despite the importance of PDGFRA signaling for OPC development, and its dysregulation in gliomas, the cellular and molecular consequences of amplified PDGFRA signaling in OPCs are unknown.

Here, we investigated the effects of overexpressing PDGFRA in murine embryonic OPCs on myelination and midline gliomagenesis. We developed a novel conditional knock‐in (KI) mouse model that overexpresses human PDGFRA (hPDGFRA) in the Rosa26 locus (Bouabe & Okkenhaug, 2013) in prenatal Olig2‐expressing progenitors. The Rosa26 locus lacks hPDGFRA's endogenous regulatory elements, including its promoter and 3′ untranslated region. This prevents the transcriptional and translational downregulation of hPDGFRA by the transcription factor Nkx2.2 (Zhu et al., 2014) and microRNAs (Dugas et al., 2010; Zhao et al., 2010), respectively, during OPC differentiation. Unexpectedly, the hPDGFRA KI mice, in the presence or absence of the tumor suppressor gene p53, failed to develop midline gliomas. The KI mice, however, exhibited severe central nervous system (CNS) hypomyelination. Examination of the transcriptome revealed negatively enriched OL gene signatures in the mutant mouse brain. Our results suggest that increased PDGFRA activity in embryonic OPCs affects OPC development leading to hypomyelination, but does not result in gliomagenesis.

1 **Significant Outcomes**

- PDGFRA overexpression in prenatal OPCs results in CNS hypomyelination.
- PDGFRA overexpression in prenatal OPCs along with p53 loss does not induce gliomas.

## 2 MATERIALS AND METHODS

### 2.1 Generation of transgenic mice

**FIGURE 1:**
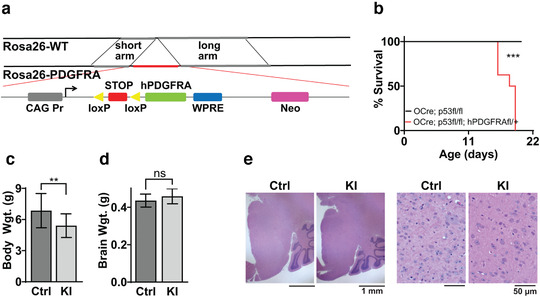
Generation and phenotype of hPDGFRA KI mice. (a) Schematic of the Rosa26 locus in control and hPDGFRA KI mice. hPDGFRA is downstream of the CAG promoter and a STOP codon flanked by loxP sites. (b) Kaplan–Meier survival curves of Olig2‐Cre; p53^fl/fl^ (Ctrl, black, median survival undefined, *n* = 11) and Olig2‐Cre; p53^fl/fl^; hPDGFRA^fl/+^ (KI, red, 18 ± 0.3651 days, *n* = 10) mice. *p* = .0001, log‐rank (Mantel–Cox) test. (c) Body weights of P18 and P19 Olig2‐Cre; p53^fl/fl^; hPDGFRA^+/+^ (Ctrl, 6.8523 ± 0.4576 g, *n* = 13) and Olig2‐Cre; p53^fl/fl^; hPDGFRA^fl/+^ (KI, 5.4088 ± 0.4024 g, *n* = 8) mice. *p* = .0431, two‐tailed unpaired Student *t*‐test. (d) Brain weights of P18 and P19 Ctrl (0.4361 ± 0.0096 g, *n* = 13) and KI (0.4591 ± 0.0143 g, *n* = 8) mice. *p* = .1811, two‐tailed unpaired Student *t*‐test. Error bars represent standard error. (e) H&E images of the brainstem (BS) of P20 Ctrl and P19 KI mice. Images representative of *n* = 4 each for Ctrl and KI

The hPDGFRA KI mice were generated using the Rosa‐CAG‐LSL‐PDGFRA targeting vector, derived from the Ai9 plasmid (gift from Hongkui Zeng, Addgene plasmid #22799; http://n2t.net/addgene:22799; RRID:Addgene_22799) (Madisen et al., 2010) by replacing *tdTomato* with hPDGFRA (gift from Dr. Eric Holland, Fred Hutchinson Cancer Research Center, Seattle WA, and Dr. Tatsuya Ozawa, National Cancer Center Research Institute, Tokyo, Japan). The vector is composed of (from 5′ to 3′) a CMV‐IE enhancer/chicken beta–actin/rabbit beta–globin hybrid promoter (CAG), an FRT site, a loxP‐flanked STOP cassette (with stop codons in all 3 reading frames and a triple polyA signal), the human *PDGFRA* cDNA, a woodchuck hepatitis virus post‐transcriptional regulatory element (WPRE, to enhance the mRNA transcript stability), a BGH polyA signal, and an attB/attP‐flanked PGK‐FRT‐Neo‐polyA cassette (Figure 1a). The linearized vector was inserted between exons 1 and 2 of the Gt(ROSA)26Sor locus via electroporation of (129 × 1/SvJ x 129S1/Sv)F1‐Kitl+‐derived R1 embryonic stem cells (Nagy et al., 1993). Homologous recombination events were detected by PCR, and correct rearrangement of the locus was verified by Southern blot analysis. Correctly targeted stem cells were injected into recipient blastocysts following standard procedures (Nagy, 2003). The resulting chimeric animals were crossed to C57BL/6J mice; heterozygotes were further crossed to C57BL/6 mice. Note that all the hPDGFRA^fl/fl^ mice used in this study are in the Nestin‐tv‐a (Ntv‐a) background, where the RCAS viral receptor TVA is downstream of the Nestin promoter (Cordero et al., 2017), but the mice were not injected with RCAS viruses. hPDGFRA^fl/fl^ mice were bred to Olig2‐tv‐a‐Cre; p53^fl/fl^ mice to generate Olig2‐tv‐a‐Cre; p53^fl/fl^; hPDGFRA^fl/+^ mice. Olig2‐tva‐Cre mice (Olig2tm2(TVA,cre)Rth/J) were obtained from the Jackson Laboratory. GFAP‐Cre mice (Zhuo et al., 2001) were a kind gift from Dr. Hai Yan, Duke University. hPDGFRA^fl/fl^ mice were crossed with GFAP‐Cre mice to generate GFAP‐Cre; hPDGFRA^fl/+^ offspring. Mutant mice were euthanized when they lost 25% of body weight, or were unable to ambulate or adequately reach food and/or water (typically seen around postnatal day 18 to 21 (P18 to P21)), along with their littermate control mice. Mice were euthanized by exposure to 2–4 liters CO_2_ per minute, then carefully decapitated to preserve brain and spinal cord (SC). To genotype hGFAP‐Cre and Olig2‐t‐va‐Cre mice, genomic tail DNA was isolated using REDExtract‐N‐Amp™ PCR ReadyMix (Sigma), and PCR was performed using the following primers: Olig2‐t‐va‐Cre – 5′ CAC AGG AGG GAC TGT GTC CT 3′, 5′ AGT TGG TGA GCA TGA GGA TG 3′, and 5′ GCC AGA GGC CAC TTG TGT AG 3′; GFAP‐Cre – transgene forward 5′ GCG GTC TGG CAG TAA AAA CTA TC 3′; transgene reverse 5′ GTG AAA CAG CAT TGC TGT CAC TT 3′; WT forward 5′ CTA GGC CAC AGA ATT GAA AGA TCT 3′; and WT reverse 5′ GTA GGT GGA AAT TCT AGC ATC ATC C 3′. hPDGFRA cDNA was extracted using a standard mouse tail DNA prep (Laird et al., 1991) and PCR run using MyFi™ Mix (Meridian Biosciences) with the following primers: WT forward 5′ AAG GGA GCG GAA AAG TCT CCA C 3′; WT reverse 5′ GGA CAA CGC CCA CAC ACC AGG 3′; and hPDGFRA – reverse 5′ TGG GCT ATG AAC TAA TGA CCC CGT A 3′.

### 2.2 In vitro studies

RCAS viruses were generated by transfecting retroviral plasmids into DF1 chicken fibroblasts (ATCC) as previously described (Barton et al., 2013). Viral supernatant was concentrated 100X with Retro‐X Concentrator (Clontech) prior to use. Primary BS cultures were generated from hPDGFRA^fl/fl^ P3–P5 pups and grown in DMEM (ATCC) supplemented with 2 mM l‐glutamine (Invitrogen), 10% FBS (ATCC), and 100U penicillin/streptomycin (Invitrogen) at 37°C. Cells collected from each litter of pups were divided into two T25 culture flasks, and at 70% confluency, they were transduced with concentrated RCAS‐Cre or RCAS‐Y viruses. Because the hPDGFRA^fl/fl^ mice are in the Ntv‐a background, the RCAS viruses are expected to target Nestin‐expressing cells (Cordero et al., 2017). Seventy‐two hours after plating, cells were serum‐starved overnight and treated with 100 nM purified PDGF‐AA ligand (Sigma) for 5 min, trypsinized, and collected by centrifugation at 1500× *g* for 5 min for western blot analysis.

### 2.3 Western blot analysis

Cultured cells, and BS and SC tissue were lysed and homogenized in RIPA buffer (Sigma) using a Q125‐125‐watt Sonicator (MedSupply) at 50% amplitude for 10 s. Tissue and cell pellets were resuspended in RIPA buffer with phosphatase inhibitor (Sigma), protease inhibitor (Roche), 1 mM PMSF (Sigma), 50 mM NaF, 1 μM NaVO_4_ (Sigma), and 1 mM DTT (Sigma). Protein concentration was determined using BCA analysis (BIORAD). Lysates were run on 4−20% Mini‐PROTEAN gel (BIORAD) at 100 V and transferred using Trans‐Blot Turbo Transfer System (BIORAD) onto Immun‐Blot PVDF membranes (BIORAD). Membranes were blocked using Intercept (PBS) Protein‐Free Blocking Buffer (LI‐COR). The following primary antibodies were used: PDGFRA (CST, 3164S), 1:1000; myelin basic protein (MBP, CST, 78896), 1:1500; phospho‐Tyr762 PDGFRA (pPDGFRA, CST, 2992), 1:1000; ERK1/2 (CST, 9107), 1:1000; phospho‐Thr202/Tyr204 ERK1/2 (pERK1/2, CST, 9101), 1:1000; Akt (CST, 2920), 1:2000; phospho‐Ser473 Akt (pAkt, CST, 4060), 1:2000; and β‐actin (CST, 3700S), 1:5000. Polyclonal goat anti‐rabbit IgG (IRDye 800CW) and goat anti‐mouse IgM (IRDye 680RD) were used as secondary antibodies. Signal intensity was measured on the Odyssey imaging system (LI‐COR) per manufacturer's instructions, and densitometry analysis done using Image Studio (LI‐COR).

### 2.4 Histology and immunohistochemistry (IHC)

Mouse brain and SC were fixed in 2% neutral buffered formalin for 24–48 h, and then transferred to 70% ethanol. Samples were embedded and sectioned by the Northwestern Mouse Histology and Phenotyping Laboratory. Sagittal sections for brain and transverse sections for SC tissue at 5 μm thickness were obtained using a Leica microtome. Upper SC (USC) segments are roughly characterized by vertebrae C4 to C7, and lower SC (LSC) by T1 to T6. Hematoxylin and eosin (H&E) staining was done according to standard protocols. Immunolabeling was performed using the Ventana automated IHC instrument (Roche) per manufacturer's instructions, and imaged on an Axiocam 503 light microscope (Zeiss) at 2.5X and 40X magnifications. The following primary antibodies were used: PDGFRA (CST, 3164S), 1:200; MBP (CST, 78896), 1:1200; 2′,3′‐cyclic‐nucleotide 3′‐phosphodiesterase (CNPase, CST, 5664), 1:100; Aspartoacylase (ASPA) (Genetex, GTX113389), 1:2000; Olig2 (Millipore, AB9610), 1:500; NG2 (Millipore, AB5320), 1:200; Sox10 (Abcam, 180862), 1:150; and Ki67 (CST, 12202S), 1:200. Nuclear staining (for Olig2, Sox10, ASPA, Ki67 antibodies) was quantified using a LionheartFX automated microscope (Biotek), and non‐nuclear staining (for NG2, MBP, CNPase antibodies) was quantified by measuring optical density (OD) (log(maximum intensity/mean intensity)) of DAB staining in deconvolved images using FIJI (Varghese et al., 2014). Similar intensity thresholds were applied for control and KI samples for each antibody, but not across antibodies. Therefore, the percentage of labeled cells across antibodies cannot be compared. Luxol fast blue (LFB) staining of brain tissue, with cresyl violet counterstain, was performed by the Northwestern University Mouse Histology and Phenotyping Laboratory. Images were captured at 2.5X and 40X magnifications.

### 2.5 RNA sequencing (RNA‐seq) and analysis

For RNA‐seq, BS was isolated from P18/P19 mice from Olig2‐Cre; p53^fl/fl^; hPDGFRA^fl/+^ and age‐matched Olig2‐Cre; p53^fl/fl^ control mice and snap frozen. RNA was extracted using RNeasy Mini Kit (QIAGEN) and quantified using Cytation5 Image Reader (BIOTEK). 50 ng of RNA was submitted to the Northwestern University Sequencing Core facility for analysis. For bioinformatics analysis, paired‐end fastq files were imported into Galaxy (Afgan et al., 2016), aligned to the mm10 genome using RNA‐STAR, and aligned reads were counted using HTSeq‐count with the Ensembl mm10 transcriptome GTF file as the feature file. The following HTSeq‐count parameters were used: stranded = no, mode = union, minimum alignment quality = 10, map nonunique or ambiguous reads = none. HTSeq‐count files were imported into R (https://www.r‐project.org/https://www.r‐project.org/), genes with < 10 reads base mean were removed, and differential expression analysis was performed with the DESeq2 package (Love et al., 2014) using the DESeqDataSetFromHTSeqCount function with default settings. DESeq2 analyses were run comparing hPDGFRA KI and control mice. Gene set enrichment analysis (GSEA) was run using genes ranked according to the Wald statistic with the following parameters: permutations = 1000, enrichment statistic = classic, max size = 500, min size = 20, normalization mode = meandiv. For the analysis in Table 2, custom GSEA gene lists were generated from (Zhang et al., 2014), by taking the top 500 genes positively expressed in astrocytes versus neurons, OPCs, microglia, newly formed OLs, myelinating OLs, or endothelial cells. Genes with minimum FPKM (fragments per kilobase per million reads) less than 5 in astrocytes were excluded. GSEA was run using the parameters described above to compare control and KI mice based on the custom gene lists. RNAseq data has been submitted to GEO (GSE181899) and are publicly‐available.

### 2.6 Experimental design and statistical analysis

Three to eleven mice of each genotype were used per experiment, with roughly equal numbers of mutant and control mice, and males and females. IHC data was quantified by analyzing one whole BS section and six SC sections spanning C4 to T6 per mouse. Unpaired, two‐tailed Student *t*‐test was used to determine statistical significance between experimental and control groups using GraphPad Prism. Kaplan–Meier survival curves were analyzed using log‐rank (Mantel‐Cox) test. *p* < .05 was considered statistically significant. The *n* and *p* values for each experiment are stated in the text and figure legends.

## 3 RESULTS

### 3.1 Generation and validation of KI mice overexpressing hPDGFRA in prenatal Olig2‐progenitors

We targeted hPDGFRA to prenatal OPCs using Olig2‐Cre mice. We inserted the WT hPDGFRA cDNA into the Rosa26 locus, allowing for the stable expression of a single transgene (Bouabe & Okkenhaug, 2013). A stop codon flanked by loxP sites is an immediate upstream of hPDGFRA, which is under the control of the CAG promoter (Figure 1a). The Rosa26 locus lacks the endogenous hPDGFRA regulatory elements, thus preventing its transcriptional (Zhu et al., 2014) and translational (Dugas et al., 2010; Zhao et al., 2010) downregulation during OPC differentiation. The tumor suppressor p53 is lost in 77% and co‐occurs with PDGFRA amplifications in 30–40% of DMG (Khuong‐Quang et al., 2012; Paugh et al., 2011); therefore, we also included p53 deletion in our mouse model. hPDGFRA^fl/fl^ mice were crossed with Olig2‐Cre; p53^fl/fl^ mice to delete p53 and express Cre recombinase in Olig2‐progenitors. Cre recombinase removes the stop codon thus expressing the hPDGFRA transgene. Deleting p53 was critical to investigate gliomagenesis in our original mouse model, but we also performed studies in models with p53 intact, as we describe later in Figures 6 and 7.

Olig2‐Cre; p53^fl/fl^; hPDGFRA^fl/+^ (KI) mice exhibited significantly decreased survival when compared with Olig2‐Cre; p53^fl/fl^ (control) mice (Figure 1b, control, mean survival undefined, *n* = 11, KI, 18 ± 0.3651 days, *n* = 10, *p* = .0001, log‐rank (Mantel–Cox) test). All the hPDGFRA KI mice (10/10) exhibited ataxia, and hindlimb and tail tremors by P18 or P19, and all the mice that survived until P21 (5/10) also exhibited hindlimb paralysis. Examination of body weights revealed stunted growth in P18 and P19 KI mice when compared with control (Figure 1c, control, 6.8523 ± 0.4576 g, *n* = 13, KI, 5.4088 ± 0.4024 g, *n* = 8, *p* = .0431, two‐tailed unpaired Student *t*‐test). The brain weights, however, were not significantly different (Figure 1d, control, 0.4361 ± 0.0096 g, *n* = 13, KI, 0.4591 ± 0.0143, *n* = 8, *p* = 0.1811, two‐tailed unpaired Student *t*‐test). H&E staining of brain sections (Figure 1e, BS is shown) revealed no gross abnormalities or gliomas. The KI mice with paralysis however exhibited hemorrhage and tissue injury in the posterior SC (not shown). Thus, prenatal overexpression of PDGFRA along with p53 loss in murine Olig2+ cells does not produce gliomas.

**FIGURE 2:**
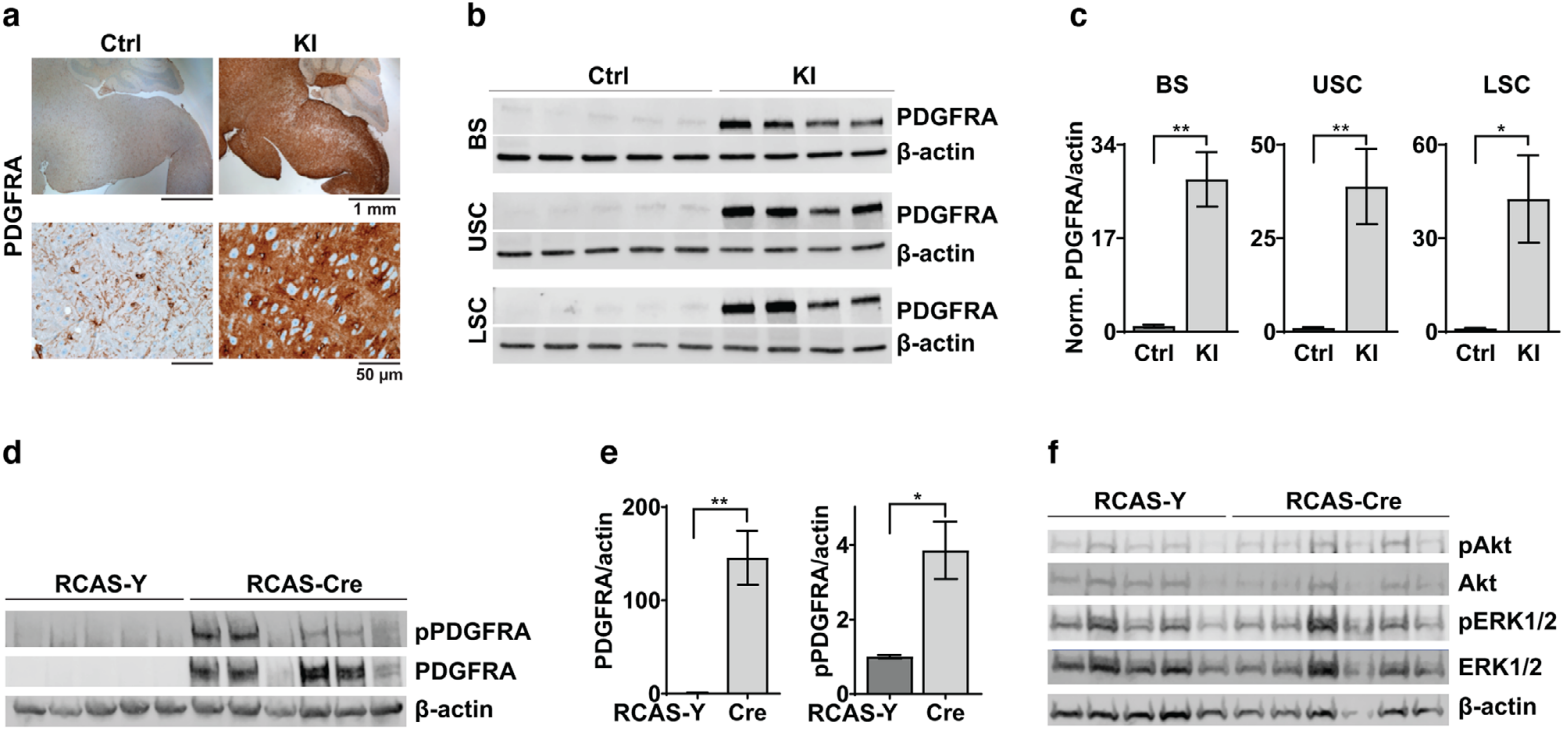
PDGFRA expression is increased in hPDGFRA KI mice. (a) IHC images of PDGFRA expression in BS of P18 Olig2‐Cre; p53^fl/fl^ (Ctrl) and Olig2‐Cre; p53^fl/fl^; PDGFRA^fl/+^ (KI) mice. Images representative of *n* = 4 each for Ctrl and KI. (b,c) Western blot images (b) and quantitation (c) of PDGFRA expression relative to β‐actin in P18 Ctrl (*n* = 5) and KI (*n* = 4) mice. BS, Ctrl 1 ± 0.2740, KI 27.7281 ± 4.9400, *p* = .0005, two‐tailed unpaired Student *t*‐test; upper spinal cord (USC, cervical to mid‐thoracic), Ctrl, 1 ± 0.2125, KI, 38.82461 ± 10.0835, *p* = .0037, two‐tailed unpaired Student *t*‐test; lower spinal cord (LSC, mid‐thoracic to lumbar), Ctrl, 1 ± 0.2070, KI, 42.6288 ± 14.0615, *p* = .0119, two‐tailed unpaired Student *t*‐test. Data are normalized to control. (d,e) Western blot images and quantitation showing expression of PDGFRA and pPDGFRA relative to β‐actin in cultured RCAS‐Y (control, n = 5) and RCAS‐Cre (KI, n = 6) BS progenitor cells. Cells were treated with viruses and PDGF‐AA. PDGFRA/β‐actin, Ctrl, 1 ± 0.31670, KI, 145.5661 ± 28.9251, p = .0014; pPDGFRA/β‐actin, Ctrl, 1 ± 0.0473, KI, 3.8570 ± 0.7691, p = .0084; two‐tailed unpaired Student t‐test. Data are normalized to control values. Error bars represent standard error. (f) Western blot images showing pAkt, Akt, pERK1/2, and ERK1/2 expression in cultured BS progenitors treated as above.

We verified that PDGFRA is indeed overexpressed in the hPDGFRA KI mice using IHC and western blot analyses. We found increased PDGFRA expression in P18 KI mouse brain when compared to control (Figure 2a, BS is shown). Western blot analysis revealed significantly elevated PDGFRA levels in BS, USC, and LSC tissue ((Figure 2b,c, BS, control, 1 ± 0.2740, *n* = 5, KI, 27.7281 ± 4.9400, *n* = 4, *p* = .0005; USC, control, 1 ± 0.2125, *n* = 5, KI, 38.82461 ± 10.0835, *n* = 4, *p* = .0037; LSC, control, 1 ± 0.2070, *n* = 5, KI, 42.6288 ± 14.0615, *n* = 4, *p* = .0119, all using two‐tailed unpaired Student *t*‐test). We further confirmed successful activation of the PDGFRA signaling pathway in cultured BS progenitors from hPDGFRA^fl/fl^ mice treated in vitro with RCAS‐Cre virus and PDGF‐AA ligand (Figure 2d,e). We found significant increases in the levels of total PDGFRA (control, 1 ± 0.31670, *n* = 5, KI, 145.5661 ± 28.9251, *n* = 6, *p* = .0014, two‐tailed unpaired Student *t*‐test) and pPDGFRA (control, 1 ± 0.0473, *n* = 5, KI, 3.8570 ± 0.7691, *n* = 6, *p* = .0084, two‐tailed unpaired Student *t*‐test) in RCAS‐Cre relative to RCAS‐Y treated cells. Levels of pAkt and pERK1/2, downstream effectors of PDGFRA, however, remained unchanged (Figure 2f, pAkt, control, 1 ± 0.2959, *n* = 5, KI, 2.6060 ± 0.7646, *n* = 6, *p* = 0.1035; pERK1/2, control, 1 ± 0.1567, *n* = 5, KI, 1.9199 ± 0.4411, *n* = 6, *p* = 0.1035, two‐tailed unpaired Student *t*‐test).

### 3.2 PDGFRA overexpression in prenatal Olig2‐progenitors results in CNS hypomyelination

**FIGURE 3:**
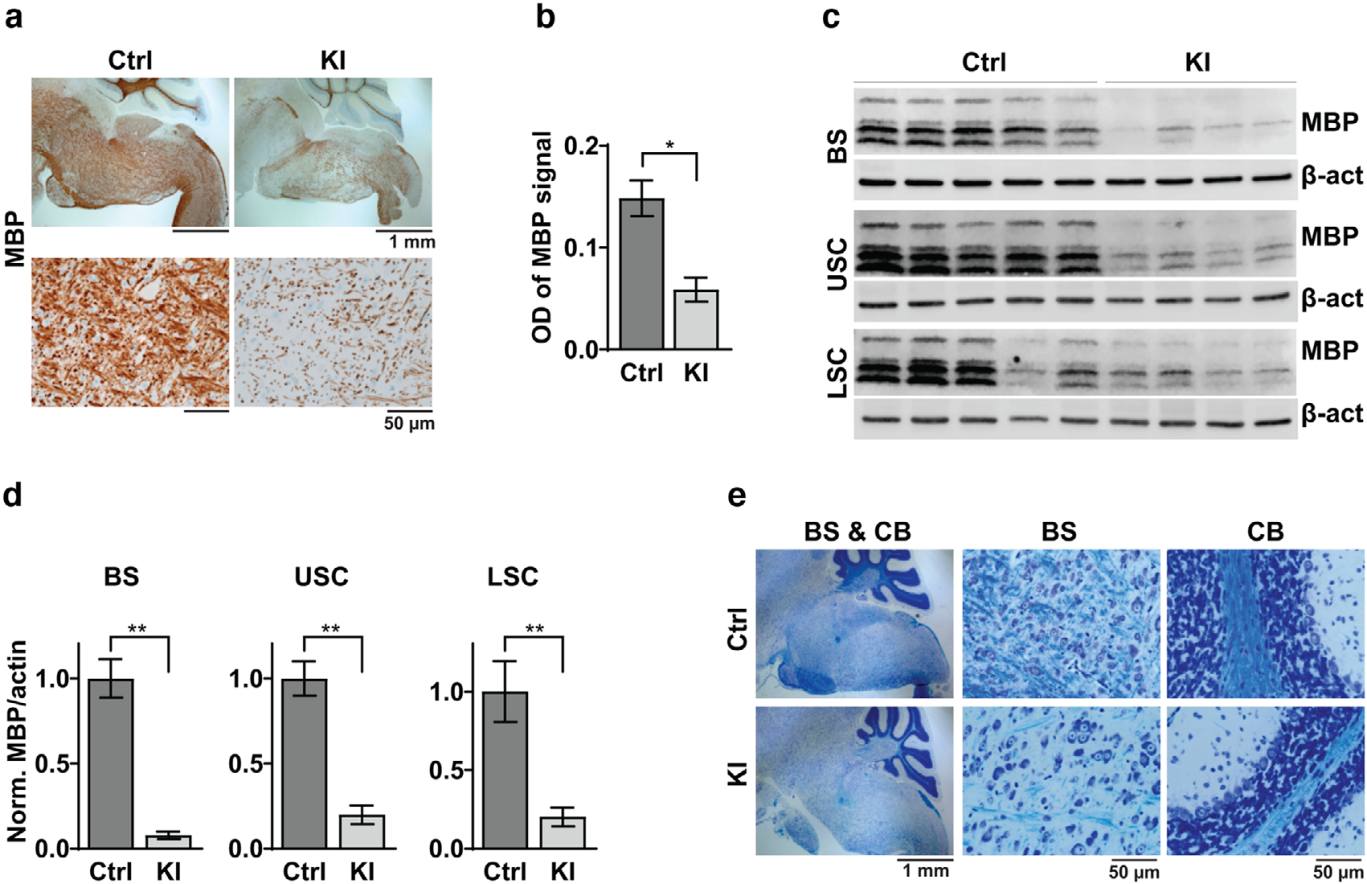
hPDGFRA KI mice exhibit reduced myelination. (a) Representative IHC images showing MBP expression in BS of P18 Olig2‐Cre; p53^fl/fl^ (Ctrl) and Olig2‐Cre; p53^fl/fl^; PDGFRA^fl/+^ (KI) mice. (b) Average optical density (OD) of MBP signal in the BS of Ctrl (0.1734 ± 0.0112, *n* = 3) and KI (0.0739 ± 0.0049, *n* = 11) mice. *p* = 8.5566E‐07, two‐tailed unpaired Student *t*‐test. (c,d) Western blot images (c) and quantitation (d) of MBP expression relative to β‐actin P19 Ctrl (*n* = 5) and KI (*n* = 4) mice. BS, Ctrl, 1 ± 0.1134, KI, 0.0786 ± 0.0222, *p* = .0002; USC, Ctrl, 1 ± 0.1005, KI, 0.19841 ± 0.0549, *p* = .0003; LSC, Ctrl, 1 ± 0.1935, KI, 0.2020 ± 0.0601, *p* = .0095; all using two‐tailed unpaired Student *t*‐test. Data are normalized to control. Error bars represent standard errors. (e) LFB stained images of P20 Ctrl and P19 KI mouse BS and cerebellum (CB). Images representative of *n* = 4 each for Ctrl and KI

The tremors and hindlimb paralysis exhibited by hPDGFRA KI mice are reminiscent of symptoms commonly seen in mouse models of hypomyelination (Calver et al., 1998; Dugas et al., 2010; Fruttiger et al., 1999; Suzuki et al., 2012; Zhu et al., 2014). Therefore, we investigated whether hPDGFRA KI mice exhibit myelin deficiencies. IHC analysis revealed a drastic loss of MBP expression in the brain (Figure 3a,b, BS is shown) of P18 hPDGFRA KI mice when compared with control (control, 0.1734 ± 0.0112, *n* = 3, KI, 0.0739 ± 0.0049, *n* = 11, *p* = 8.5566E‐07, two‐tailed unpaired Student *t*‐test). We confirmed this observation with western blot analysis of P19 BS and SC tissue (Figure 3c,d, BS, control, 1 ± 0.1134, *n* = 5, KI, 0.0786 ± 0.0222, *n* = 4, *p* = .0002; USC, control, 1 ± 0.1005, *n* = 5, KI, 0.19841 ± 0.0549, *n* = 4, *p* = .0003; LSC, control, 1 ± 0.1935, *n* = 5, KI, 0.2020 ± 0.0601, *n* = 4, *p* = .0095, all using two‐tailed unpaired Student *t*‐test). Further, qualitative evaluation of LFB‐stained (Carriel et al., 2017) brain tissue sections revealed substantial global hypomyelination in KI mice when compared to control (Figure 3e, BS and cerebellum (CB) are shown). Overall, the above results suggest that prenatal overexpression of hPDGFRA in Olig2‐progenitors leads to CNS hypomyelination in mutant mice.

### 3.3 PDGFRA overexpression decreases oligodendroglial and myelination gene signatures

**FIGURE 4:**
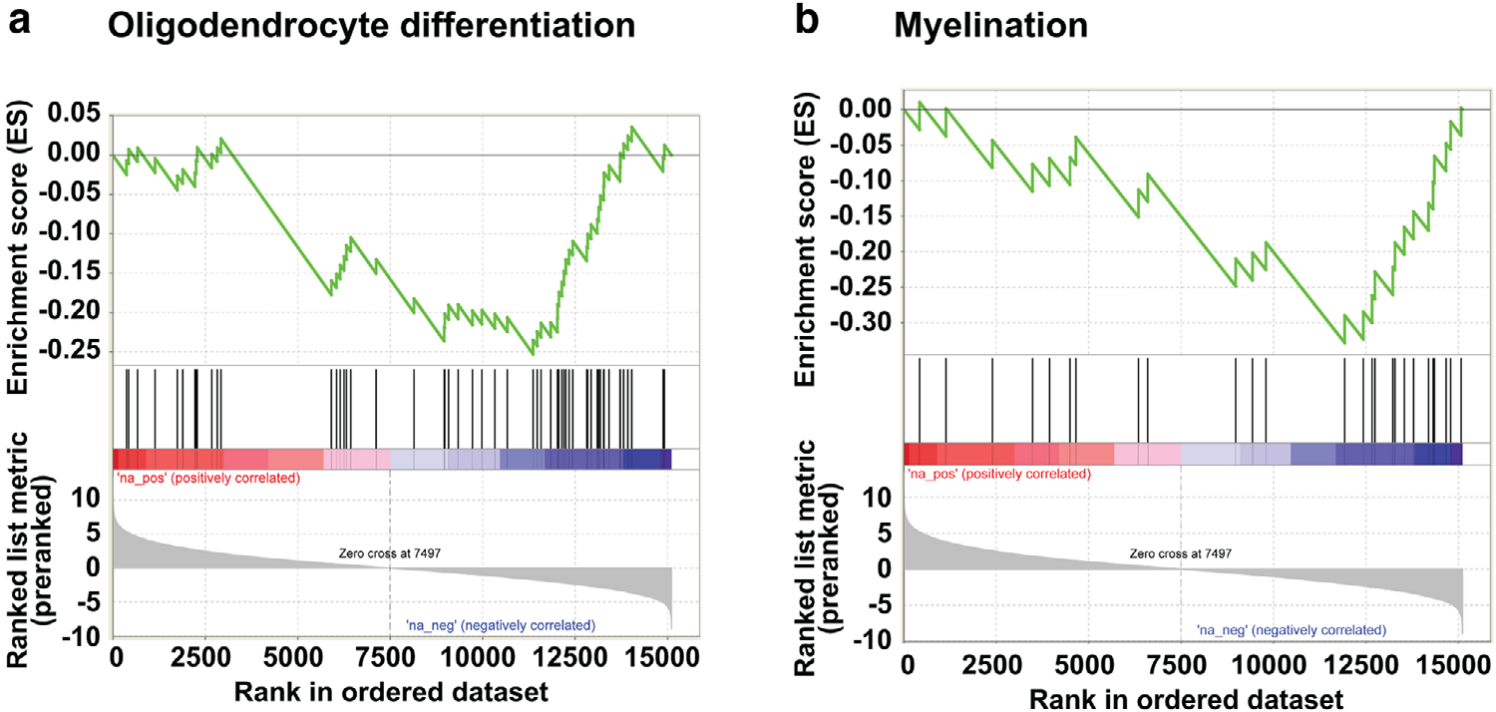
Oligodendrocyte and myelination signatures are reduced in hPDGFRA KI mice. (a,b) GSEA plots showing decreased representation of OL differentiation (a) and myelination (b) gene signatures in P19 Olig2‐Cre; p53^fl/fl^; PDGFRA^fl/+^ (*n* = 4) mice when compared with Olig2‐Cre; p53^fl/fl^ (*n* = 4) mice

To identify the molecular changes accompanying hypomyelination in mice overexpressing hPDGFRA, we performed RNA‐seq analysis of BS tissue derived from P19 Olig2‐Cre; p53^fl/fl^; hPDGFRA^fl/+^ and Olig2‐Cre; p53^fl/fl^ mice. Using a false discovery rate (FDR) of ≤0.05, we identified >5000 significantly differentially expressed genes in the hPDGFRA KI mice, >2600 of which were significantly downregulated. Importantly, several OL lineage markers were downregulated in the KI mice as shown in Table 1, consistent with the myelination defects described in Figure 3. GSEA revealed decreased representation of OL differentiation (Figure 4a) and myelination (Figure 4b) gene signatures in the KI mice. Intriguingly, transcripts for several astrocytic markers were increased (Table 1). Since astrocytic markers are few and not highly specific to astrocytes, we performed GSEA using gene lists comparing astrocytes with other cell types in the brain (Zhang et al., 2014). This analysis revealed positive enrichment of astrocytic signatures relative to several other cell types in the KI mice (Table 2). We also observed approximately a three‐fold increase in *PDGFRA* transcript levels in the KI mice compared to control (KI, 8337, *n* = 4, control, 2907, *n* = 4, *p* = .0004, FDR adjusted). We also aligned the FASTQ files to the human genome and found hPDGFRA expression in the KI samples but not in the controls (exon 22, KI, 674, *n* = 4, control, 36, *n* = 4, *p* = .04, two‐tailed unpaired Student *t*‐test). These data validate the hPDGFRA KI mice, along with the IHC and western blot results shown in Figure 2. Thus, PDGFRA overexpression in prenatal Olig2‐progenitors decreases OL lineage and myelination gene signatures, and enriches astrocytic gene signatures.

**TABLE 1:** List of differentially expressed markers of oligodendroglial and astrocytic lineages in hPDGFRA KI mice compared to control

Cell type	Gene name	Log2 fold change	FDR Adj. *p*‐value
OL lineage	*CSPG4/NG2*	−1.13	.0003*
	*OLIG1*	−0.81	.0025*
	*OLIG2*	−0.86	.0051*
	*SOX10*	−0.80	.0140*
	*NKX2.2*	−0.60	.0188*
	*MYRF*	−0.81	.0121*
	*CNP*	−0.74	.0244*
	*MOG*	−0.71	.0272*
	*MOBP*	−0.54	.0569
	*PLP1*	−0.57	.0745
	*MBP*	−0.32	.3049
Astrocytes	*GLUL*	0.72	.0019*
	*SOX9*	0.47	.0086*
	*APOE*	0.36	.0203*
	*S100β*	0.41	.0296*
	*GFAP*	0.32	.3715

RNA‐seq data from BS of P18/P19 Olig2‐Cre; p53^fl/fl^; PDGFRA^fl/+^ (*n* = 4) and Olig2‐Cre; p53^fl/fl^ (*n* = 4) mice.

* *p* < .05.

**TABLE 2:** Astrocytic gene signatures are enriched relative to other cell types in hPDGFRA KI mice

Astrocyte vs.	Size	ES	NES	NOM *p*‐val	FDR *q*‐val	FWER *p*‐val
Newly formed OL	462	0.266086	6.631515	0	0	0
Myelinating OL	453	0.224306	5.620024	0	0	0
OPC	460	0.22091	5.39882	0	0	0
Neuron	466	0.251861	6.300609	0	0	0
Microglia	466	0.202658	5.059472	0	0	0
Endothelial	453	0.201959	4.962529	0	0	0

Abbreviations: ES, enrichment score; FDR, false discovery rate; FWER, family wise error rate; NES, normalized enrichment score; NOM, nominal.

RNA‐seq data from BS of P18/P19 Olig2‐Cre; p53^fl/fl^; PDGFRA^fl/+^ (*n* = 4) and Olig2‐Cre; p53^fl/fl^ (*n* = 4) mice. “0” indicates value <.01.

**FIGURE 5:**
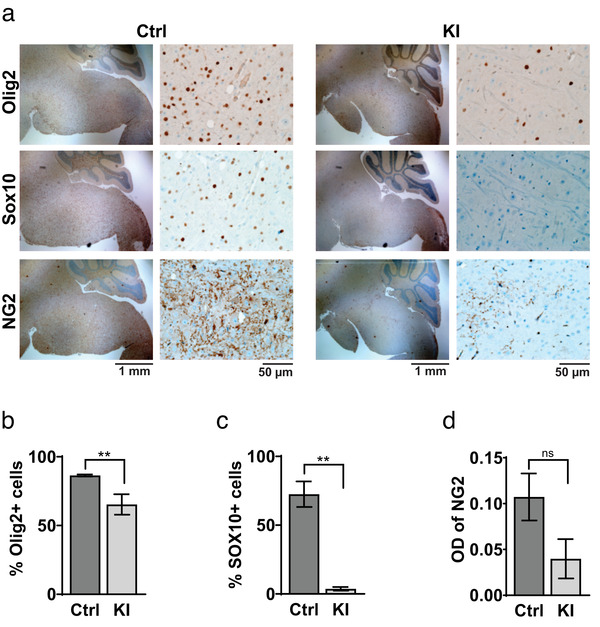
PDGFRA overexpression decreases OPC population. (a) Representative IHC images showing expression of Olig2, Sox10, and NG2 expression in BS of P19‐P20 Olig2‐Cre; p53^fl/fl^ (Ctrl) and Olig2‐Cre; p53^fl/fl^; PDGFRA^fl/+^ (KI) mice. (b–d) Quantification of Olig2, Sox10, and NG2 expression. Olig2, Ctrl, 52.3700 ± 9.4416, *n* = 3, KI, 16.3286 ± 2.7616, *n* = 11, *p* = .0002; Sox10, Ctrl, 17.6810 ± 3.8759, *n* = 4, KI, 0.6496 ± 0.4042, *n* = 5, *p* = .0016; NG2, Ctrl, 0.1072 ± 0.0257, *n* = 4, KI, 0.0398 ± 0.0215, *n* = 4, *p* = .0904; all using two‐tailed unpaired Student *t*‐test. Error bars represent standard errors

The significant decrease in transcript levels of important OPC genes such as Olig2, NG2, and Sox2 (Table 1) suggests a reduction in the OPC population in the hPDGFRA KI mice. We confirmed this using IHC analysis. We found a significant reduction in the expression level of two commonly used OPC markers Olig2 (Nishiyama et al., 1999) (Figure 5a,b, control, 52.3700 ± 9.4416, *n* = 3, KI, 16.3286 ± 2.7616, *n* = 11, *p* = .0002, two‐tailed unpaired Student *t*‐test) and Sox10 (Figure 5a,c, control, 17.6810 ± 3.8759, *n* = 4, KI, 0.6496 ± 0.4042, *n* = 5, *p* = .0016, two‐tailed unpaired Student *t*‐test) in hPDGFRA KI mice when compared to control. We also observed a reduction in expression of another common OPC marker NG2, although the difference was not statistically significant (Figure 5a,d, control, 0.1072 ± 0.0257, *n* = 4, KI, 0.0398 ± 0.0215, *n* = 4, *p* = .0904, two‐tailed unpaired Student *t*‐test) in the BS and SC (not shown) of P19‐P20 hPDGFRA KI mice. We obtained similar results in SC tissue from hPDGFRA KI mice (not shown). Thus, mice overexpressing hPDGFRA in prenatal Olig2‐progenitors exhibit a significant reduction in the OPC population by weaning age.

### 3.4 Hypomyelination in hPDGFRA KI mice is independent of p53 loss

**FIGURE 6:**
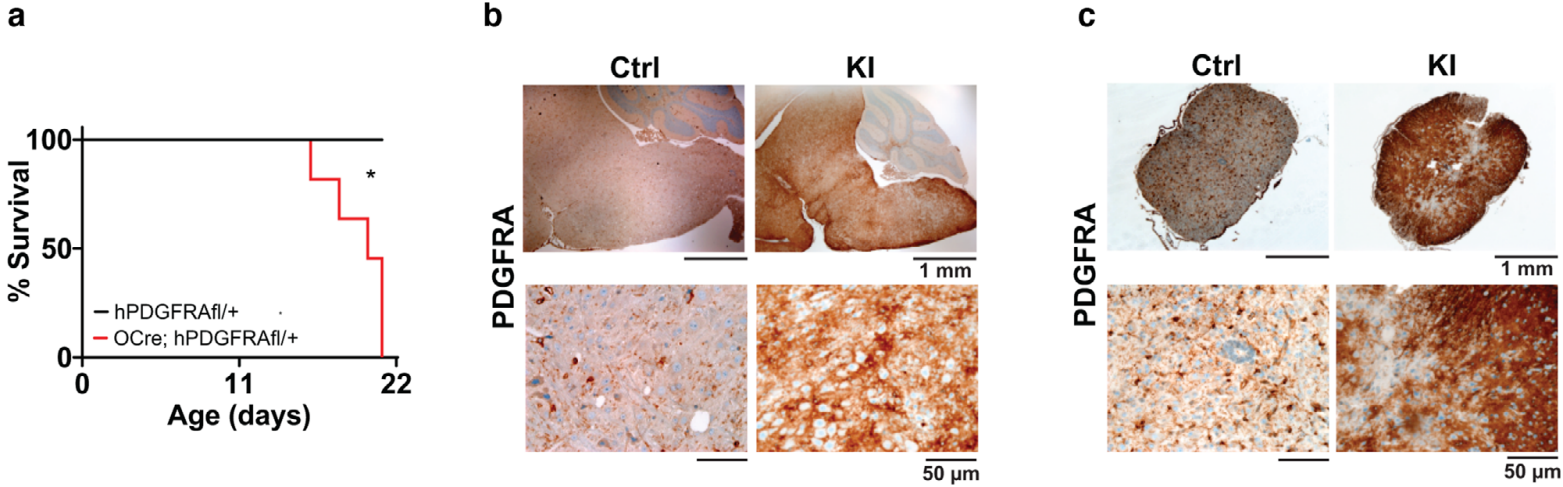
Validation of hPDGFRA KI mice without p53 loss. (a) Kaplan–Meier survival curves of hPDGFRA^fl/+^ (Ctrl, black, median survival undefined, *n* = 9), and Olig2‐Cre; hPDGFRA^fl/+^ (KI, red, 20 ± 0.6071 days, *n* = 11) mice. *p* = 0010, log‐rank (Mantel–Cox) test. (b,c) IHC images of PDGFRA expression in BS (b) and SC (c) of P21 Ctrl and P20 KI mice. Images representative of *n* = 3 each for Ctrl and KI

The above studies were carried out in hPDGFRA KI and control mice that also lack expression of the tumor suppressor p53 because of our initial investigations of glioma formation in these mice. We were interested in examining whether p53 loss was necessary for the development of myelination defects. We generated Olig2‐Cre; hPDGFRA^fl/+^ (KI) mice and compared its phenotype with control hPDGFRA^fl/+^ mice, both in p53 WT backgrounds. The KI mice exhibited ataxia, tremors, and hindlimb paralysis and were euthanized by P21 (Figure 6a, control, mean survival undefined, *n* = 9, KI, 20 ± 0.6071, *n* = 11, *p* = .0010, log‐rank (Mantel–Cox) test). The KI mice were significantly stunted in growth (control, 9.1933 ± 0.4735, *n* = 6, KI, 7.1738 ± 0.2230, *n* = 8, *p* = .0012, two‐tailed unpaired Student *t*‐test), though their brains appeared slightly larger than control (control, 0.4314 ± 0.0104, *n* = 6, KI, 0.5088 ± 0.0041, *n* = 8, *p* = .0011, two‐tailed unpaired Student *t*‐test). We confirmed increased expression of PDGFRA in the BS (Figure 6b) and SC (Figure 6c) of P20‐P21 KI mice relative to control. No gross abnormalities were detected in the brain tissue of KI mice, using H&E staining (not shown), but hemorrhaging was seen in SC tissue, similar to the phenotype observed in Olig2‐Cre; p53^fl/fl^; hPDGFRA^fl/+^ mice described earlier. Further, the percentage of Ki67+ cells in BS tissue was not significantly different between control and KI mice at P0 (control, 0.5053 ± 0.1759, *n* = 3, KI, 0.4523 ± 0.2675, *n* = 3; 0.8765, two‐tailed unpaired Student *t*‐test), P10 (control, 9.0707 ± 7.1957, *n* = 3, KI, 2.9860 ± 1.4948, *n* = 3; *p* = 0.4543, two‐tailed unpaired Student *t*‐test), or P19 (control, 0.0230 ± 0.0180, *n* = 3, KI, 0.0417 ± 0.0101, *n* = 3; *p* = 0.4170, two‐tailed unpaired Student *t*‐test), supporting the absence of neoplastic transformation in the hPDGFRA KI mice (Figure S1).

**FIGURE 7:**
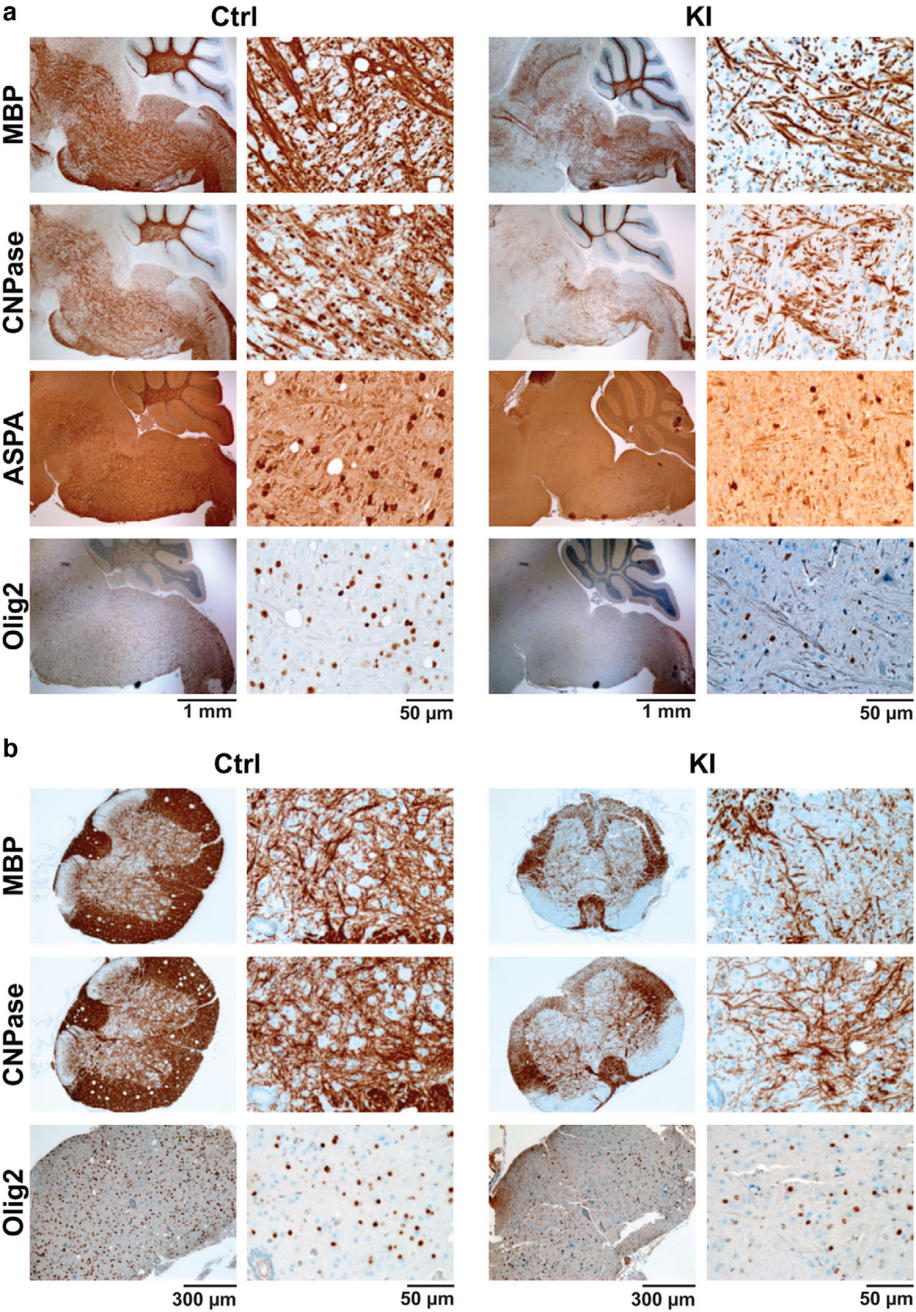
hPDGFRA KI mice exhibit hypomyelination without p53 loss. (a) Representative IHC images of MBP, CNPase, ASPA, and Olig2 expression in the BS of P21 hPDGFRA^fl/+^ (Ctrl) and Olig2‐Cre; hPDGFRA^fl/+^ (KI) mice. (b) IHC images of MBP, CNPase, and Olig2 expression in the USC of P21 Ctrl and KI mice. Quantitation of MBP, CNPase, ASPA, and Olig2 IHC data for BS and SC is provided in Table 3

hPDGFRA KI mice without p53 loss, however, exhibited a significant reduction in the expression of MBP, CNPase, and ASPA, markers of mature myelinating OLs, in P19‐21 KI BS and SC when compared with control (Figure 7a,b and Table 3). This is similar to the loss of myelination markers seen in Olig2‐Cre; p53^fl/fl^; hPDGFRA^fl/+^ mice (Figures 3 and 5). MBP and CNPase expression levels were not significantly different between control and KI at P0, but progressively declined with postnatal development in the KI, and were significantly lower than the control at P19–P21 in both the BS and the SC (Table 3). We also observed a similar progressive reduction in Olig2‐expressing cells in the KI BS and SC versus control (Figure 7a,b and Table 3). Thus, hPDGFRA overexpression in prenatal Olig2‐expressing progenitors results in hypomyelination in the brain and SC, with or without p53 loss.

**TABLE 3:** Expression of OL markers in hPDGFRA KI mice with no p53 loss

	Brainstem			Spinal cord		
Age	Ctrl	KI	*p*‐Value	Ctrl	KI	*p*‐Value
MBP						
P0	0.0202 ± 0.0029 (*n* = 10)	0.0257 ± 0.0037 (*n* = 6)	.2598	0.0013 ± 0.0003 (*n* = 8)	0.0017 ± 0.0008 (*n* = 7)	.6447
P10	0.0541 ± 0.0064 (*n* = 9)	0.0408 ± 0.0035 (*n* = 7)	.1149	0.0171 ± 0.0028 (*n* = 9)	0.0195 ± 0.0034 (*n* = 8)	.5707
P19	0.1734 ± 0.0112 (*n* = 3)	0.0751 ± 0.0049 (*n* = 11)	2.4852 e‐06	0.1968 ± 0.0175 (*n* = 3)	0.0520 ± 0.0069 (*n* = 7)	2.9716 e‐05
CNPase						
P0	0.0362 ± 0.0026 (*n* = 10)	0.0327 ± 0.0031 (*n* = 6)	.4148	0.0133 ± 0.0013 (*n* = 3)	0.0129 ± 0.006 (*n* = 4)	.7476
P10	0.0506 ± 0.0085 (*n* = 9)	0.0391 ± 0.0037 (*n* = 7)	.2830	0.0873 ± 0.0123 (*n* = 8)	0.0469 ± 0.0062 (*n* = 8)	.0110
P19	0.1379 ± 0.0033 (*n* = 3)	0.0546 ± 0.0044 (*n* = 11)	.0091	0.1929 ± 0.0117 (*n* = 3)	0.0620 ± 0.0059 (*n* = 7)	3.417e‐06
ASPA						
P19	81.3960 ± 7.2522 (*n* = 3)	45.4767 ± 6.2821 (*n* = 3)	.0201			
Olig2						
P0	23.1292 ± 7.0806 (*n* = 6)	53.9793 ± 15.4279 (*n* = 6)	.0992	29.5640 ± 5.5861 (*n* = 10)	22.8110 ± 4.6375 (*n* = 5)	.4487
P10	34.7171 ± 4.1461 (*n* = 7)	67.4031 ± 7.2442	.0024	79.6210 ± 2.7749 (*n* = 7)	69.3078 ± 3.2994 (*n* = 6)	.0345
P19	52.3700 ± 9.4416 (*n* = 3)	16.3286 ± 16.3286 (*n* = 11)	.0002	32.9730 ± 6.7569 (*n* = 6)	10.7081 ± 2.5569 (*n* = 9)	.0035

Values represent OD for MBP, CNPase, and ASPA, and percentage positive cells for Olig2, obtained by quantifying IHC images from BS and SC of P0, P10, and P19‐P21 hPDGFRA^fl/+^ (Ctrl) and Olig2‐Cre; hPDGFRA^fl/+^ (KI) mice. *p* values were determined using two‐tailed unpaired Student *t*‐test.

### 3.5 PDGFRA overexpression in prenatal GFAP progenitors leads to hypomyelination

**FIGURE 8:**
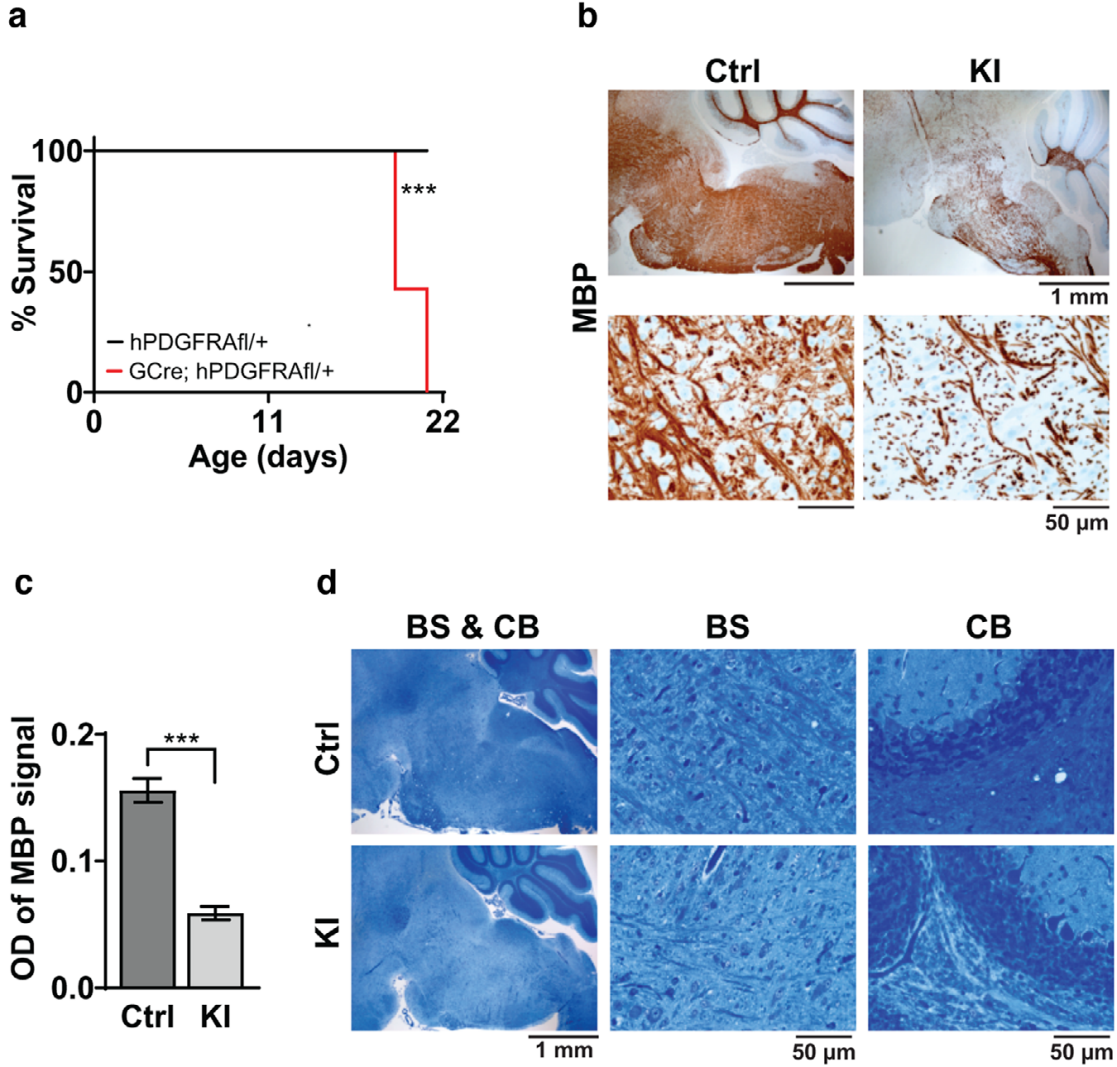
Prenatal overexpression of hPDGFRA in GFAP‐progenitors leads to hypomyelination. (a) Kaplan–Meier survival curves of hPDGFRA^fl/+^ (Ctrl, black, median survival undefined, *n* = 13), and GFAP‐Cre; hPDGFRA^fl/+^ (KI, red, 19 ± 0.2745 days, *n* = 13) mice. *p* = .0001, long‐rank (Mantel–Cox) test. (b,c) IHC images (b) and quantitation (c) of MBP OD in the BS of P19 Ctrl and P20 KI mice. Error bars represent standard errors. (d) LFB stained images of P19 Ctrl and P20 KI mouse BS and CB. Images representative of *n* = 7 each for Ctrl and KI

To investigate whether overexpression of hPDGFRA in a wider compartment of cells than OPCs gives rise to hypomyelination, we crossed hPDGFRA^fl/fl^ mice with GFAP‐Cre mice. GFAP‐progenitors are not specific to OPCs, instead give rise to a more diverse lineage of cells including neurons and glia (Semerci & Maletic‐Savatic, 2016). GFAP‐Cre; hPDGFRA^fl/+^ KI mice, but not hPDGFRA^fl/+^ control mice, exhibited ataxia and tremors and were euthanized by P21 (Figure 8a, control, mean survival undefined, *n* = 13, KI, 19 ± 0.2745 days, *n* = 13, *p* = .0001, log‐rank (Mantel–Cox) test). The KI mice developed myelination defects, supported by reduced MBP expression (Figure 8b,c, control, 0.1556 ± 0.0095, *n* = 8, KI, 0.0589 ± 0.0053, *n* = 8, *p* = 3.719E‐07, two‐tailed unpaired Student *t*‐test) and LFB staining (Figure 8d) when compared to control. These findings are similar to those observed with Olig2‐Cre; hPDGFRA^fl/+^ KI mice shown in Figure 7. Thus, prenatal overexpression of WT hPDGFRA in Olig2‐ or GFAP‐expressing progenitors leads to CNS hypomyelination.

## 4 DISCUSSION

PDGF signaling promotes OPC maintenance and proliferation, but inhibits OPC differentiation into myelinating OLs (Butt, Hornby, Ibrahim, et al., 1997; Butt, Hornby, Kirvell & Berry, 1997; Ellison & de Vellis, 1994; Hall et al., 1996; Hart et al., 1989; Pringle et al., 1989; Richardson et al., 1988). *PDGFRA* gene copy number variations and amplifying mutations are found in 4–39% of pediatric DMG, whose cell‐of‐origin is thought to be OPCs (Filbin et al., 2018; Lindquist et al., 2016; Nagaraja et al., 2017). However, the in vivo consequences of increased PDGFRA expression in OPCs has not been described before. In this study, we analyzed the consequences of overexpression of WT hPDGFRA in prenatal Olig2‐expressing OPCs. The mutant mice exhibited severe hypomyelination in the brain and SC. Overexpressing hPDGFRA more globally using GFAP‐Cre also resulted in CNS myelination defects. hPDGFRA overexpression with or without p53 loss, however, did not result in tumors. Our results suggest that prenatal hPDGFRA overexpression in glial progenitors leads to defective oligodendroglial development and hypomyelination in the CNS.

We overexpressed hPDGFRA in embryonic Olig2‐ and the more ubiquitously expressed GFAP‐progenitors (Zhuo et al., 2001) using the Rosa26 locus that lacks the endogenous regulatory elements of *hPDGFRA*. We show that the hPDGFRA KI mice exhibit severe hypomyelination in the CNS (Figures 3, 7, and 8), which explains the stunted growth, ataxia, and early mortality seen in the mutant mice. We observed 100% mortality in the hPDGFRA KI mice driven by Olig2‐Cre or GFAP‐Cre by weaning age (Figures 1, 6, and 8). Importantly, total PDGFRA levels were significantly higher in the mutant brain and SC tissue compared with control indicating successful overexpression of hPDGFRA (Figures 2, 6, and 8). We also confirmed PDGFRA phosphorylation in cultured BS progenitors from hPDGFRA^fl/fl^ mice, treated in vitro with RCAS‐Cre and PDGF‐AA (Figure 2). Activation of the PDGFRA effectors Akt and ERK1/2 was not seen, possibly because of the short incubation periods, 5 min (Figure 2) and 10 min (not shown), with the ligand. It is also possible that activation of these molecules is acute and was not captured in western blot analysis.

Examination of the transcriptome revealed reduced OL differentiation and myelination gene signatures in Olig2‐Cre; p53^fl/fl^; hPDGFRA^fl/+^ mouse brains relative to Olig2‐Cre; p53^fl/fl^ control (Figure 4 and Table 1). Consistent with that observation, we found decreased expression of the mature oligodendroglial markers MBP, CNPase, and ASPA in the hPDGFRA KI mouse brain and SC (Figures 3, 7, 8, and Table 3). Defects in myelination were also apparent in LFB stained images of the brain (Figures 3 and 8). Importantly, expression of Olig2 and Sox10, markers of OPCs, appeared normal at P0 and P10 but were significantly reduced by weaning age in the KI brain and SC suggesting a defect in OPC development (Figures 5, 7, and Table 3). The reduction in OPCs could be either due to increased apoptosis as reported for mice overexpressing PDGF‐A (Calver et al., 1998) and/or due to differentiation into other lineages such as astrocytes, which is supported by our RNA‐seq data (Tables 1 and 2). In fact, loss of Olig2 in NG2+ OPCs has been shown to increase differentiation into astrocytes at the expense of OLs (Zuo et al., 2018), and Olig2 deletion converts gliomas from proneural to astrocytic signatures (Lu et al., 2016). Myelination defects were seen in the hPDGFRA KI mice both in the presence and the absence of p53, indicating that the mutational status of p53 does not impact hypomyelination.

Our results are consistent with previous reports suggesting that PDGFRA downregulation is important for OPC differentiation. Conditional deletion of PDGFRA in Olig1+ OPCs leads to decreased OPC proliferation but premature OPC differentiation resulting in hypomyelination, consistent with PDGFRA's dual role in promoting OPC proliferation but inhibiting OPC differentiation (Zhu et al., 2014). Overexpressing the PDGFRA inhibitory transcription factor Nkx2.2 also results in precocious OPC differentiation (Zhu et al., 2014). On the other hand, disrupting the generation of PDGFRA inhibitory factors, such as microRNAs, affects OPC differentiation and myelination (Dugas et al., 2010). Mice that overexpress the PDGF‐A ligand exhibit a transient increase in the embryonic OPC population that returns to baseline levels in postnatal mice (Calver et al., 1998). Both the hPDGFRA KI mice, described here, and PDGF‐A KO mice (Fruttiger et al., 1999) exhibit hypomyelination, which suggests a developmental balancing act where too little or too much of PDGF‐A/PDGFRA signaling is deleterious to OL development. Of note, hypomyelination was not reported in mice overexpressing PDGF‐A (Calver et al., 1998). The authors reported increased OLs in late embryonic and neonatal mutant mice when compared to control, due to increased OPC proliferation, but the OLs return to baseline levels by P6. The lack of myelination defects with PDGF‐A overexpression could be because PDGFRA downregulation and, therefore, normal OPC differentiation into OLs, remains unaffected in these mice. The authors also did not look past P6. In our study, MBP and CNPase levels are not significantly different between control and KI mice at P0 or P10, but only at P19 (Table 3). In addition, unlike the PDGF‐A overexpressing mice that show increased OPCs (PDGFRA+) at embryonic and neonatal stages relative to control, our PDGFRA KI mice do not show increased OPCs (Olig2+) at P0, possibly because PDGF‐A levels are limiting (Calver et al., 1998) and excess PDGFRA does not result in increased OPC proliferation.

PDGFRA expression in the developing mouse CNS is high until approximately P14, corresponding temporally with proliferation and migration of glial progenitors, and then decreases in the adult brain (Yeh et al., 1993). Thus, the progressive myelination defects seen in hPDGFRA KI mice (Table 3) may be attributed to the high level of PDGFRA that is maintained into adulthood, supporting the importance of downregulation of PDGFRA signaling for normal OPC differentiation. It is also possible that hPDGFRA overexpression affects the later stages of myelination such as myelin sheath maintenance instead of OPC differentiation or myelin initiation, as shown for mice lacking FGF receptors in OL‐lineage cells (Furusho et al., 2012). *Fgfr1/Fgfr2* ablation in CNP+ or Olig1+ cells lowers the number of myelin transcription genes in late adulthood, but not in early myelination. The mutant mice have normal sheath initiation but disproportionally thin sheath later in adulthood (Furusho et al., 2012). Further, the continued expression of myelination regulatory genes is important for maintaining sheath integrity post‐initiation, and myelin regulatory factor ablation in mature OLs results in delayed CNS myelination (Koenning et al., 2012). The exact mechanism by which PDGFRA overexpression impacts OL development, including effects on OPC proliferation, differentiation, apoptosis, and myelin sheath maintenance, and PDGFRA's interplay with other growth factors and myelin regulatory genes will be evaluated in future studies.

A surprising finding of our study is that PDGFRA overexpression in embryonic glial progenitors, with or without p53 loss, did not result in tumors. Possible reasons for the lack of gliomas are discussed below. First, Olig2‐Cre and GFAP‐Cre transgenes are activated early in embryonic development (∼ E13.5) (Takebayashi et al., 2002; Zhuo et al., 2001). Sustained high levels of PDGFRA expression driven by these transgenes, in multiple cell types, could induce neurodevelopmental perturbations preventing gliomagenesis in our models. Second, PDGFRA amplification may not be a precipitating event in tumorigenesis, supported by the observation that activating PDGFRA mutations but not WT PDGFRA initiate gliomas in the context of p53 loss in mice (Paugh et al., 2013). The tumorigenic impact of WT PDGFRA may also be context‐dependent as PDGFRA overexpression with *Ink4a/Arf* tumor suppressor deletion promotes gliomagenesis (Liu et al., 2011). PDGF‐A and PDGF‐B, although rarely amplified in the human disease (Paugh et al., 2010; Puget et al., 2012; Zarghooni et al., 2010), consistently induce gliomas in mouse models (Hambardzumyan et al., 2009; Misuraca et al., 2015; Ozawa et al., 2014), even with no accompanying oncogenic mutations (Becher et al., 2010). This discrepancy could be due to the non‐cell autonomous activation of PDGF signaling in endothelial or stromal cells by PDGF ligands, and/or differences in the timing of pathway activation (prenatal vs. neonatal mice). Third, if PDGFRA alterations are a late event in human tumors, they may require earlier founding events to exert tumorigenic effects. Thus, further investigations are needed to determine the potential tumorigenic impact of WT PDGFRA in the context of alternative genetic alterations, cells‐of‐origin, and/or timing of genetic perturbations.

A potential caveat of the Olig2‐Cre driven hPDGFRA KI mouse model is that Olig2‐progenitors in the ventral neuroepithelium first give rise to motor neurons (MNs) (∼ E10‐E13), followed by OPCs (Fu et al., 2002; Takebayashi et al., 2002; Wu et al., 2006), raising the possibility that the hypomyelination in the KI mice is a result of MN abnormalities. We consider this possibility unlikely, given that inspection of H&E stained images did not show gross abnormalities in the MN distribution in the SC. Due to the ubiquity of transgene expression with GFAP‐Cre, and the fact that Olig2‐progenitors also give rise to MNs, future studies could utilize PDGFRA‐Cre mice to overexpress the PDGFRA transgene specifically in OPCs (Fruttiger et al., 1999), and match the spatiotemporal expression patterns of endogenous PDGFRA. It is also possible that prenatal overexpression of PDGFRA, regardless of the promoter used, is deleterious for neurodevelopment. Therefore, an inducible system such as CreER, or CNPase‐Cre, could help activate PDGFRA later in development to examine myelin dysregulation.

In conclusion, prenatal hPDGFRA overexpression in Olig2‐ or GFAP‐expressing progenitors disrupts the normal development of the oligodendroglial lineage, leading to severe CNS hypomyelination. Our PDGFRA KI mice may represent a novel model for investigating hypomyelinating disorders and treatments.

## CONFLICT OF INTEREST

The authors declare no competing financial interests.

## AUTHOR CONTRIBUTIONS

Herminio Joey Cardona, Agila Somasundaram, Donna M. Crabtree, and Oren J. Becher designed research. Herminio Joey Cardona performed experiments and the majority of the data analysis. Samantha L. Gadd performed bioinformatics analysis. Agila Somasundaram, Herminio Joey Cardona, and Donna M. Crabtree wrote the manuscript with input from the other authors. Oren J. Becher edited the manuscript and supervised the project. All authors read and approved the final version of the manuscript.

### PEER REVIEW

The peer review history for this article is available at https://publons.com/publon/10.1002/brb3.2332

## Supporting information



Supporting Information

## Data Availability

The data that support the findings of this study are available from the corresponding author upon reasonable request.
